# From Innovation to Integration: Bridging the Gap Between IoMT Technologies and Real-World Health Management Systems

**DOI:** 10.3390/s25216660

**Published:** 2025-11-01

**Authors:** Sara Jayousi, Chiara Barchielli, Sara Guarducci, Marco Alaimo, Stefano Caputo, Paolo Zoppi, Lorenzo Mucchi

**Affiliations:** 1 PIN Foundation, Prato Campus of University of Florence, 59100 Prato, Italy; 2 Management and Health Laboratory, Institute of Management, Sant’Anna School of Advanced Studies of Pisa, 56127 Pisa, Italy; chiara.barchielli@santannapisa.it; 3 Department of Information Engineering, University of Florence, 50139 Florence, Italy; sara.guarducci@unifi.it (S.G.); stefano.caputo@unifi.it (S.C.); lorenzo.mucchi@unifi.it (L.M.); 4 Department of Nursing and Midwifery, Local Health Unit Toscana Centro, 50121 Florence, Italy; marco.alaimo@uslcentro.toscana.it (M.A.); paolo.zoppi@uslcentro.toscana.it (P.Z.)

**Keywords:** Information and Communication Technologies, healthcare, patients’ management, Internet of Medical Things, health monitoring

## Abstract

This study lays the foundation for a multidimensional framework aimed at facilitating the effective integration of Internet of Medical Things (IoMT) technologies into real-world health management systems. It critically examines the technological, organizational, and societal barriers that hinder this transition and identifies key enabling conditions, such as interoperability, user co-design, and ethical design principles, that promote sustainability, inclusiveness, and trust. By proposing a structured approach to integration, this paper aims to bridge the gap between innovation and long-term, reliable adoption across diverse healthcare contexts.

## 1. Introduction

### 1.1. Background and Motivation

The rapid evolution of the Internet of Medical Things (IoMT) and Wireless Sensor Networks (WSNs) is revolutionizing the healthcare landscape. These technologies enable continuous monitoring, personalized care delivery, and remote support, offering promising solutions to the growing needs of aging populations and individuals with chronic or complex conditions [1,2]. From wearable devices and telemedicine platforms to home automation systems and artificial intelligence (AI)-driven analytics, the spectrum of tools available today has the potential to transform the way health and social care are accessed, coordinated, and delivered [3,4]. However, the true value of these innovations can only be realized when they are effectively integrated into real-world care ecosystems, tailored to the dynamic needs of individuals and local communities [5].

### 1.2. From Innovation to Integration

Despite the proliferation of advanced technologies, the healthcare sector continues to face a significant gap between innovation and real-world adoption. This divide stems from the coexistence of fragmented, heterogeneous solutions; a lack of interoperability and shared standards; and insufficient alignment with actual territorial and user-specific needs [6,7]. Moreover, digital literacy remains a critical barrier; many end users, both patients and professionals, are not adequately trained to adopt and use these technologies effectively [8,9]. Information and Communication Technologies (ICts) should not be seen as standalone tools but as enablers of integrated, user-centered ecosystems where care is personalized, coordinated, and sustainable. Bridging the gap requires not only technological innovation but also strategic governance, training for user acceptance, and systemic integration efforts [10,11].

### 1.3. Scope and Contributions

This paper aims to bridge the critical gap between technological innovation and the effective, sustainable integration of Internet of Medical Things (IoMT) solutions into real-world health management systems. While the literature extensively addresses the technical advancement of IoMT, fewer studies offer a comprehensive, interdisciplinary roadmap for their systemic adoption within public healthcare infrastructures.

A critical and interdisciplinary overview of the challenges and opportunities associated with the real-world integration of IoMT technologies is provided by analyzing both technical and socio-organizational barriers. We propose a strategic framework for the user-centered deployment of IoMT systems that are context-aware, ethically grounded, and operationally scalable.

The paper is organized as follows: after providing the background, motivation, scope, and key contributions in Section 1, Section 2 presents an overview of the proposed framework, explaining its structural organization. Section 3, Section 4, Section 5 and Section 6 each elaborate on one of the four core axes of the framework, addressing key dimensions such as technological landscape, integration barriers, strategic methods for sustainable implementation, and equity and governance considerations. Section 7 illustrates the practical application of these axes within the context of a case study. Finally, Section 8 concludes the paper by summarizing the main insights and outlining future directions.

## 2. Framework Overview

The aim of this section is to give an overview of the framework defined to articulate a strategic pathway for the adoption of IoMT within real health systems. By surveying what already exists in the wild and curating applications and possibilities, we offer scholars and practitioners a navigational aid, a multidimensional framework that functions as a “movable” SWOT (Strengths, Weaknesses, Opportunities, and Threats) analysis, to guide research and practice across heterogeneous contexts. In line with our overarching proposition—“a strategic framework for the user-centered deployment of IoMT systems that are context-aware, ethically grounded, and operationally scalable”—the novelty of our contribution lies in bridging fragmented knowledge and enabling its systematic application in practice.

Operationally, the framework is organized along four interdependent axes: (i) technological landscape, (ii) integration barriers, (iii) enabling strategies for sustainable integration, and (iv) equity, sustainability, and governance. Each axis functions both as an analytical lens and as a practical lever, enabling stakeholders to adapt the framework to specific use cases, organizational settings, or territorial ecosystems. Below, each axis and its primary outputs are summarized as follows:

1. **Axis 1—Technological Landscape Assessment.** Establishes an evidence-based map of the current IoMT ecosystem: core components (sensors, WBANs, and gateways), enabling technologies (AI/ML, edge–cloud architectures, and XR), typical data flows, and proven application domains (acute care, home monitoring, and rehabilitation). This assessment identifies maturity levels, integration gaps, and emergent capabilities, producing a technology inventory, maturity matrix, and prioritized RWD needs to guide selection and procurement.
2. **Axis 2—Systematic Classification of Integration Barriers.** Provides a structured diagnosis of adoption obstacles across four domains: technological (interoperability, explainability, validation, and security); organizational (workflow fit, training, and resource allocation); ethical/regulatory (privacy, consent, liability, and compliance); and human-centered (usability, trust, and adherence). The classification yields targeted barrier profiles and risk registers that inform mitigation planning and pilot design.
3. **Axis 3—Strategic Methods for Sustainable Integration.** Translates diagnostics into actionable strategies and tools: participatory co-design and stakeholder engagement, digital literacy and on-boarding programs, regulatory and validation pathways (clinical evaluation, Medical Device Regulation, and GDPR alignment), and adaptive infrastructures (HL7 FHIR middleware, and edge–cloud orchestration). This axis also specifies operational artefacts (interoperability middleware, explainability frameworks, validation pipelines, and training curricula) to support scalable, ethically grounded deployments.
4. **Axis 4—Equity, Sustainability, and Governance Analysis.** Embeds inclusion, long-term engagement, economic sustainability, and accountable governance into integration pathways. Key deliverables include equitable access models (hybrid digital/analog approaches), cost-effectiveness and budget-impact analyses, multi-stakeholder governance bodies (digital steering committees, data stewardship arrangements), and alignment with policy instruments (privacy law, AI regulation, and health data spaces).

Taken together, these four axes form a flexible, iterative framework that supports readiness assessment, strategy formulation, implementation monitoring, and continuous improvement. Applied as a movable SWOT matrix, its use generates real-world data (RWD) that feed improvement cycles and strengthen economic and policy justification for IoMT investments.

**Figure 1:**
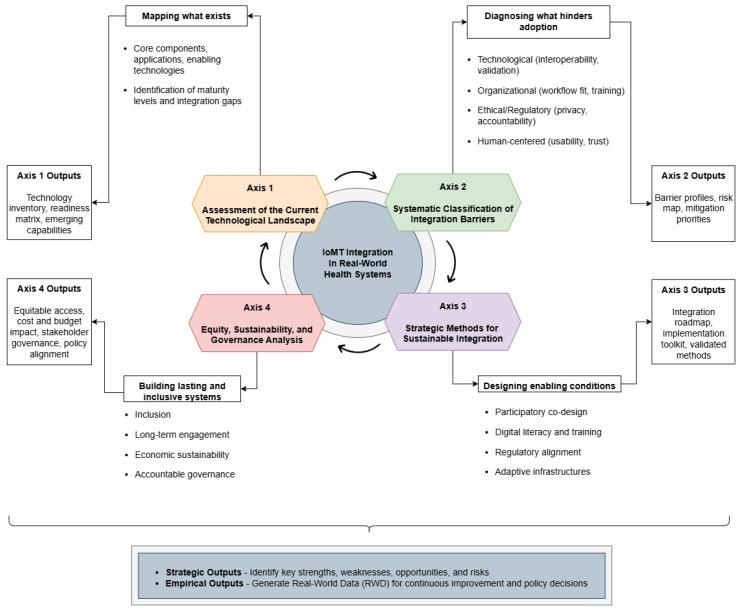
Conceptual overview of the framework structure.

The following subsections provide a detailed exposition of each axis, including conceptual foundations, operational steps, and suggested instruments for practical application. The overall structure of the proposed framework and the interrelations among its four axes are illustrated in Figure 1, which provides a conceptual overview of its organization.

## 3. Axis 1: Technological Landscape Assessment

### 3.1. Core IoMT Technologies

The IoMT constitutes a rapidly advancing technological paradigm that integrates a wide range of inter-network-connected medical devices specifically designed for healthcare monitoring and management. These devices facilitate autonomous, real-time health surveillance by leveraging a combination of automated systems, advanced sensor interfaces, and machine learning-driven AI. Through secure network infrastructures, IoMT technologies establish a continuous communication channel between patients and healthcare providers, enabling remote acquisition, processing, and transmission of critical medical data [12].

At the core of this interconnected ecosystem lie WSNs and Wireless Body Area Networks (WBANs), which enable the continuous monitoring of physiological parameters. WSNs are typically composed of spatially distributed sensor nodes that collaboratively gather and relay biomedical data—such as body temperature, arterial blood pressure, oxygen saturation levels, and electrocardiographic signals—across a networked environment [13,14]. WBANs, a specialized subclass of WSNs, are designed to function in close proximity to or within the human body. They offer high-resolution, real-time data acquisition capabilities while maintaining ultra-low latency and minimal energy consumption [15,16]. These networks are crucial in enabling pervasive health monitoring, particularly in ambulatory care and home-based medical settings, where continuous oversight is essential for proactive and personalized healthcare delivery [16].

In parallel, AI and Machine Learning (ML) algorithms have emerged as indispensable in analyzing and interpreting the vast and complex data streams generated by IoMT systems. These technologies facilitate a range of advanced functionalities such as pattern recognition, predictive modeling, and decision support, which are essential for converting raw sensor outputs into clinically relevant information [17,18]. For example, ML algorithms can be trained to recognize early manifestations of cardiac arrhythmias by analyzing electrocardiogram (ECG) patterns [19], or to anticipate hypoglycemic or hyperglycemic episodes in diabetic patients by analyzing historical glucose levels alongside contextual variables such as nutritional intake and physical activity [20]. The integration of AI/ML not only improves the accuracy and timeliness of diagnostic processes, but also facilitates the implementation of personalized medicine by tailoring therapeutic interventions to the unique physiological and behavioral profiles of individual patients [21].

Another essential element within the IoMT framework is the emergence of digital biomarkers, which refer to objectively measurable physiological and behavioral metrics captured through digital technologies. These metrics serve as indicators for normal biological functions, pathological conditions, or biological responses triggered by environmental exposures or therapeutic interventions [22]. Unlike traditional biomarkers, which are typically measured in controlled clinical environments at discrete time points, digital biomarkers can be continuously monitored in real-world settings, offering enhanced temporal resolution and contextual relevance [23]. They are increasingly being used in areas such as neurodegenerative disease monitoring, mental health assessment, and oncology [24,25,26].

Finally, Extended Reality (XR), an umbrella term that includes virtual reality, augmented reality, and mixed reality, is emerging as a transformative element within the healthcare technology landscape. These immersive technologies are reshaping how digital content is experienced and interacted with, offering new dimensions of engagement, visualization, and simulation [27]. As XR continues to evolve, it holds the potential to significantly influence the way healthcare professionals, patients, and digital systems interact, fostering more intuitive, responsive, and integrated care environments [28,29].

### 3.2. Current Applications

The practical implementation of IoMT technologies now extends across a wide range of healthcare contexts. Local health authorities have participated in pioneering case studies that exemplify how such technologies can be seamlessly integrated into diverse healthcare environments, particularly within the constraints and complexities of a publicly funded health system [30]. The widespread integration of IoMT technologies not only in clinical settings but also in home-based environments highlights their flexibility, scalability, and increasing technological maturity. As these systems become more embedded in routine healthcare operations, they are driving a transition toward preventive, data-informed, and patient-centric models of care, applicable in both institutional frameworks and remote settings [31].

In clinical environments, IoMT solutions are widely adopted for continuous and automated patient monitoring, particularly in high-dependency units such as intensive care, neonatal wards, and post-operative recovery areas [32,33,34]. Wearable sensors and implantable devices enable real-time tracking of vital parameters—such as heart rate, respiratory rate, oxygen saturation, and blood pressure—allowing clinicians to detect early signs of physiological deterioration and intervene promptly [35,36,37]. These systems are often integrated with hospital information systems and electronic health records (EHRs), facilitating seamless data flow and supporting clinical decision-making [38]. Additionally, connected medical devices such as smart infusion pumps, ventilators, and telemetry systems contribute to the delivery of precision medicine by ensuring accurate, responsive, and individualized care [39,40,41].

In primary care and outpatient settings, IoMT is increasingly coupled with AI-driven diagnostic platforms to enhance the accuracy and efficiency of clinical assessments. Advanced imaging systems equipped with ML algorithms assist in the detection of various pathologies, including dermatological lesions, pulmonary abnormalities, and retinal disorders [42,43,44]. These tools can process large volumes of data in seconds, highlighting potential anomalies and supporting clinicians in prioritizing cases that require further evaluation. In several domains, such as diabetic retinopathy and skin cancer detection, AI-enhanced diagnostics have demonstrated performance comparable to or exceeding that of experienced specialists [45,46].

In the context of home-based care, IoMT plays a pivotal role in enabling remote patient monitoring, particularly for individuals managing chronic conditions such as heart failure, chronic obstructive pulmonary disease, and diabetes mellitus [47,48,49]. A wide range of interconnected devices—including smart glucometers, digital blood pressure monitors, and wearable ECG patches—enables the real-time transmission of physiological data to healthcare professionals. This continuous flow of information allows for timely adjustments to treatment plans, early detection of complications, and improved patient adherence, while also reducing the need for in-person consultations and hospital admissions [50,51,52]. For instance, studies [53] have documented the deployment of ECG-enabled wearable devices connected to cloud-based decision support systems, showing promising outcomes such as significant reductions in unplanned readmissions and emergency department visits. Notably, these systems often integrate AI-driven triage mechanisms, which assist healthcare providers by prioritizing alerts on clinician dashboards, thereby facilitating more efficient patient outreach and resource allocation. In addition, mobile health applications are being employed in mental health monitoring, capturing behavioral and physiological indicators such as sleep patterns, physical activity, and mood fluctuations, which can serve as early warning signs for conditions like depression and anxiety [54]. In the context of asthma management, especially in pediatric populations, mobile applications are used to monitor inhaler use and symptom patterns, as well as to identify environmental triggers through integration with geolocation data [55,56].

The field of rehabilitation is also undergoing significant advancements through the integration of IoMT. In particular, XR platforms are increasingly being employed to deliver customized rehabilitation programs, allowing patients to engage in therapeutic exercises remotely while being monitored and guided through virtual supervision [57,58]. A key feature of these platforms is the incorporation of gamification elements or inclusive strategies, which are designed to boost patient motivation, engagement, and adherence to prescribed regimens by transforming repetitive exercises into interactive and rewarding experiences [59,60].

### 3.3. Demonstrated Benefits

The integration of IoMT technologies into modern healthcare systems has produced a range of demonstrated benefits, as highlighted by findings from diverse research efforts, practical applications, and real-world deployments. Among the most notable advantages is the significant enhancement of operational efficiency. By automating the collection of clinical data and enabling continuous, real-time patient monitoring, IoMT solutions alleviate the workload traditionally carried by healthcare professionals. This, in turn, allows clinicians to focus more time and attention to direct patient care [61]. For example, automated systems for monitoring vital signs have been shown to reduce the frequency of manual assessments, minimize documentation errors, and streamline workflows within hospital settings [62,63].

Another key benefit is the improvement of clinical outcomes. A growing body of research has demonstrated that remote monitoring technologies facilitate earlier detection of medical complications, support more effective disease management, and contribute to a reduction in hospital readmissions [64]. In patients with heart failure, for instance, the use of IoT-based devices for remote monitoring has been linked to a measurable decrease in emergency department visits and hospitalizations [49]. Additionally, AI-driven diagnostic tools have enhanced both the accuracy and speed of disease identification, enabling more timely and targeted therapeutic interventions [65].

IoMT also plays a crucial role in advancing personalized medicine [66,67]. By leveraging continuous data streams and AI analytics, clinicians can tailor interventions to the unique physiological and behavioral characteristics of individual patients. This personalized approach is particularly valuable in managing chronic diseases, where care plans must be dynamically adjusted in response to evolving patient needs [68]. Personalized rehabilitation programs, adaptive medication dosing, and context-aware behavioral interventions are just a few examples of how IoMT supports individualized care [69,70,71].

Furthermore, IoMT technologies play a significant role in enhancing patient engagement and empowerment. Through wearable devices and mobile health applications, individuals are enabled to take an active role in managing their own health. These tools allow patients to continuously monitor key health indicators, track progress over time, and maintain communication with healthcare professionals. This proactive involvement has been consistently linked to improved adherence to prescribed treatment regimens, greater health literacy, and higher levels of patient satisfaction [72,73].

Finally, from a broader perspective, IoMT has the potential to enhance the sustainability and resilience of healthcare systems. By enabling the decentralization of care, optimizing resource utilization, and supporting proactive, data-driven interventions, IoMT supports a more distributed and responsive model of care. These capabilities are particularly valuable in addressing systemic challenges, including shortages in the healthcare workforce, rising healthcare costs, and the need ensure continuity and coordination of care across both routine operations and emergency situations [74].

## 4. Axis 2: Systematic Classification of Integration Barriers

The integration of IoMT technologies into healthcare systems faces several multidimensional barriers. These challenges span technological, organizational, ethical, regulatory, and human-centered domains and collectively hinder the sustainable and scalable deployment of digital health solutions. Table 1 provides an overview of the key barriers categories, which are explored in more detail in the following subsections.

### 4.1. Technological Integration, Interoperability, and Explainability

The real-world adoption of IoMT systems continues to face significant challenges, many of which stem from infrastructural fragmentation and limited interoperability across heterogeneous platforms. Despite growing enthusiasm for digital transformation in healthcare, the current ecosystem remains fragmented, with devices and systems often relying on proprietary protocols and incompatible data formats [75]. This lack of standardization hinders seamless data exchange, limits scalability, and obstructs the development of integrated care pathways. Furthermore, the absence of shared standards complicates the organization of different providers (medical, social, and caregiving) who must collaborate to support fragile individuals.

Efforts to standardize communication protocols and data models are therefore essential to enable modular integration and ensure that technological innovation translates into meaningful improvements in care delivery. Pilot initiatives across European and national contexts, such as IoF2020, Synchronicity, ACTIVAGE, and U4IoT, have demonstrated the feasibility of linking physical infrastructure upgrades with interoperable digital platforms and adaptive clinical routines [30]. However, many healthcare systems still rely on outdated digital infrastructures, including isolated electronic health record (EHR) silos and fragmented local area networks (LANs), which are incompatible with the high-throughput, low-latency, and edge–cloud capabilities required by modern IoMT systems [31,75].

These infrastructural limitations not only disrupt clinical workflows but also highlight the need for coordinated investments in digital infrastructure, workflow redesign, and workforce training. Yet, even where infrastructure is in place, several critical research gaps continue to hinder the full realization of IoMT’s potential.

One such gap is the underdevelopment of real-time explainability in AI-driven healthcare applications. While AI adoption is accelerating, most current models prioritize retrospective analysis over immediate, actionable feedback. In clinical settings where timely decisions are vital, there is a pressing need for AI systems that can provide transparent, real-time explanations for alerts, predictions, and recommendations. This includes designing user-facing interfaces that translate complex algorithmic outputs into clinically meaningful insights, tailored to the cognitive needs of different stakeholders (e.g., physicians, nurses, and patients).

Another emerging challenge lies in the deployment of AI on edge devices, such as local sensors and diagnostic machines. While edge computing offers advantages in latency reduction, privacy protection, and bandwidth efficiency, it also introduces new complexities in trust and validation. Traditional centralized validation methods are insufficient in these decentralized environments. New frameworks are needed to assess reliability, detect performance drift, and ensure security over time. Promising approaches include local anomaly detection, federated learning validation, and context-aware risk scoring based on diverse data sources [76,77].

Finally, the integration of IoMT systems into clinical workflows and patients’ daily routines remains an ongoing challenge. Many current solutions are rigid, relying on static configurations that struggle to adapt to dynamic conditions, such as fluctuations in a patient’s health status, environmental changes, or varying clinical workloads. Future research should focus on developing adaptive systems capable of responding to real-time contextual changes. This includes leveraging situational awareness models, incorporating human feedback loops, and intelligently prioritizing alerts and interventions.

### 4.2. Workflow and Organizational Capacity

The integration of the IoMT into modern healthcare systems has revealed significant infrastructural limitations and incompatibilities with traditional clinical workflows. Technical integration alone is not sufficient to ensure the successful deployment of IoMT solutions. A key determinant of success lies in the alignment of clinical workflows and the organizational capacity to adapt [75,78].

When workflows are rigid and training is inadequate, systemic friction can arise. As highlighted in [78], physicians experienced cognitive overload when required to navigate two separate interfaces (standard EHR and IoMT dashboards). This dual-interface burden contributed to alert fatigue and increased the average clinical time per patient by nearly 15%. These challenges are further addressed by [75], who advocate for the co-design of workflows that seamlessly incorporate real-time patient-generated health data (PGHD) into existing clinical dashboards.

Therefore, from an organizational perspective, participatory co-design methodologies engaging healthcare professionals, patients, and system engineers seem necessary to align digital tools with existing clinical practices and mitigating alert fatigue [79,80,81]. Integrating IoMT data streams into unified EHR dashboards facilitates seamless data visualization, reduces cognitive strain, and improves clinical situational awareness among clinicians [81,82]. Furthermore, structured digital literacy programs and ongoing professional education have been linked to increased confidence and higher adoption rates of IoMT technologies among both healthcare providers and patients [83]. Indeed, institutions that fail to provide targeted training reported IoMT adoption rates below 25%, whereas those that invest in continuous professional development achieve adoption rates exceeding 60% within six months [75].

### 4.3. Ethical, Legal, and Regulatory Constraints

As healthcare systems progressively digitize through the deployment of IoMT, new ethical, legal, and regulatory complexities arise, requiring an integrated framework that aligns innovation with compliance, patient autonomy, and societal trust [84]. The first among the regulatory concerns is data privacy. Under the European Union’s General Data Protection Regulation (GDPR), Regulation (EU) 2016/679), healthcare data are classified as “special categories” requiring heightened protection. Patient Generated Health Data (PGHD), especially those collected through wearable and remote sensors, produce continuous, health data streams often gathered outside formal clinical settings. This continuous collection process challenges traditional informed consent mechanisms, data minimization norms, and purpose limitation principles—particularly when data are stored in the cloud or shared with third-party analytics providers [85].

To address these concerns, local health authorities in Italy have adopted a hybrid data governance model in which sensitive sensor data are locally anonymized through edge computing nodes before transmission to central health repositories. This architecture enables these institutions to remain compliant with national and European data protection regulations, particularly the GDPR, while ensuring secure and efficient data integration for clinical and administrative use. This architectural design ensures that only de-identified or pseudonymized data traverse public or cloud infrastructures, in alignment with GDPR Articles 25 and 32 concerning data protection by design and default [86].

Security concerns also intersect with these regulatory issues. Poorly integrated IoMT endpoints can become vectors for cyberattacks, threatening core hospital operations. Secure architectures—such as segmented VLANs and secure boot protocols—are essential to mitigate these risks [87].

Ethical considerations extend to transparency, explainability, and fairness. Trust in IoMT systems hinges on explainability: patients and clinicians alike must understand how device algorithms generate risk scores or recommendations [88]. Moreover, the obligation to ensure equity and inclusivity intersects with ethics and law. Bias in training datasets can yield predictive disparities, disproportionately affecting underrepresented patient populations [89]. Liability frameworks constitute an additional regulatory frontier. If a therapeutic recommendation derived from an IoMT device leads to adverse outcomes, determining fault—between device manufacturers, algorithm developers, and healthcare providers—remains legally ambiguous [84].

### 4.4. User Acceptance, Transparency, and Trustworthiness

The long-term adoption of IoMT systems relies not only on their technical robustness, but also on their ability to integrate seamlessly into users’ everyday lives and clinical routines [90,91]. Usability allows researchers to evaluate how easy it is to use a system, as well as how functionality is related to both the task and the person performing it [92]. Recent studies underscore that user acceptance is shaped by a combination of factors, including device comfort, intuitive usability, and perceived clinical value [91,93].

Ensuring patient adherence becomes especially crucial when sensors are worn continuously or require active user engagement [94]. In such contexts, devices that are cumbersome, intrusive, or uncomfortable can markedly reduce compliance over time. The assessment of usability is even more pronounced in populations with cognitive or physical impairments [95,96,97], where even minimally invasive technologies may be rejected due to discomfort, anxiety, or limited understanding of their purpose.

To enhance acceptance, IoMT systems should be designed with human-centered principles, prioritizing miniaturization, automation, and seamless integration into everyday routines. Studies emphasize that usability and low user burden are essential for sustained engagement [98]. Moreover, transparency and trustworthiness are foundational to user confidence. Trust in IoMT systems—whether from patients, caregivers, or clinicians—relies on transparent system behavior, including clarity about the data being collected and the information presented. Equally important is the explainability of critical decisions, such as alerts or therapeutic recommendations, and the guidance provided on what actions or tasks users are expected to perform in response [93]. Striking a balance between clinical-grade accuracy and human-centered usability is essential to ensure that these technologies are not only effective, but also adopted, accepted, and sustained over time.

## 5. Axis 3: Strategic Methods for Sustainable Integration

The real-world integration of IoMT technologies requires more than technical deployment. It depends on a set of coordinated strategies that encompass infrastructure, governance, user engagement, and regulatory readiness. Table 2 summarizes the main potential strategies and enabling tools identified. Each is explored in the following subsections. Topics related to digital equity and accessibility are introduced here but elaborated in Section 6.1.

### 5.1. Contextual Adaptation and Participatory Design: Aligning Technology with Clinical and Social Settings

The sustainable advancement of IoMT systems depends not only on technological innovation but also on inclusive, context-aware design and governance. Effective integration of IoMT technologies into healthcare systems requires alignment with the specific clinical, organizational, and socio-cultural environments in which they are implemented. This alignment is particularly crucial in public health systems, where operational workflows, patient demographics, and institutional mandates often differ significantly from those in private or academic settings. Technologies that fail to account for existing workflows, professional roles, and institutional routines risk being underutilized or rejected, regardless of their technical merit [99].

To address this risk, adaptation strategies must begin with a thorough analysis of local care pathways and the digital maturity of the healthcare environment. Ensuring interoperability with existing information systems, minimizing disruption to healthcare professionals, and embedding technologies within real clinical scenarios are essential components of this process [99].

Critical is the adoption of participatory design methodologies that actively involve end-users—clinicians, patients, caregivers, and administrators—in the design, testing, and refinement of IoMT solutions. This collaborative approach not only improves the relevance and usability of these technologies but also fosters a sense of ownership among stakeholders. Moreover, co-design practices facilitate the early identification of usability issues, uncover contextual constraints, and promote interdisciplinary dialogue. Thus, to fully harness the potential of IoMT, it is essential to integrate expertise from engineering, clinical practice, behavioral sciences, and ethics. Such interdisciplinary collaboration enables the development of solutions that are technically robust, user-centered, and ethically grounded. As recent studies have emphasized [5,11], this integration is a prerequisite for innovation that is both impactful and sustainable. Furthermore, participatory approaches that include a broader range of actors—such as social workers and community representatives—can further enhance user engagement and support long-term adoption [10]. Complementing these efforts, impact assessment frameworks rooted in public health and social sciences provide the analytical foundation necessary to inform policy decisions and guide investment strategies [100]. These tools help ensure that technological progress aligns with the public interest and contributes to equitable, value-driven transformation of health systems.

In conclusion, interdisciplinary collaboration is not optional, it is imperative. The transformation of health systems through IoMT requires a holistic approach that values technical excellence alongside ethical integrity, social relevance, and user-centered design. Only through such integrated efforts can IoMT technologies meaningfully contribute to the evolution of healthcare systems [101,102].

### 5.2. Enabling Platforms and Infrastructures

The deployment of IoMT systems in real-world healthcare environments demands more than technical connectivity: it requires architectures capable of dynamically balancing latency, interoperability, regulatory compliance, and operational cost. While previous sections outlined the functional requirements, here we analyze the enabling infrastructural paradigms, trade-offs, and applied integration models.

Edge–cloud architectures have emerged as a critical enabler for responsive healthcare applications. Edge nodes, positioned close to the data source, for example in hospital wards or patient homes, allow for a near real-time signal processing, reducing both network congestion and dependency on continuous cloud connectivity. This is especially advantageous in time-critical use cases, such as the detection of cardiac arrhythmias, the detection of falls, or automated insulin dosing [4,103]. However, edge deployments introduce heterogeneity and distributed attack surfaces, demanding additional orchestration layers and continuous validation mechanisms. Hybrid models that dynamically allocate computation between edge and cloud, based on clinical urgency and network conditions, are increasingly being adopted to balance cost, energy efficiency, and clinical responsiveness.

Interoperability middleware is essential to bridge diverse medical devices, electronic health records (EHRs), and clinical decision support systems [31,75]. Standards such as HL7 FHIR, IEEE 11073, and OpenEHR provide semantic harmonization, yet real-world deployments often require custom mapping to local data ontologies and terminologies. Middleware not only supports data exchange but also underpins modularity, enabling incremental upgrades without disrupting existing workflows. The trade-off lies in balancing strict conformance (which maximizes compatibility) with implementation pragmatism (which minimizes integration cost) [31,75]. At the application layer, well-documented open APIs (e.g., RESTful and gRPC) enable external developers to access and extend platform functionalities while supporting fine-grained access control and auditability, essential for both regulatory compliance and operational transparency [3,11].

Security-by-Design and Lifecycle Governance are indispensable in distributed IoMT ecosystems. Zero-trust network architectures, hardware-backed encryption, VLAN segmentation, secure boot protocols, and mutual authentication frameworks mitigate risks associated with data interception and unauthorized access [3,87]. Anyway, incorporating frequent security updates into regulated medical environments remains challenging. Here, DevSecOps pipelines, combined with regulatory-compliant versioning, allow continuous integration of patches without invalidating device certification, though this requires close coordination with notified bodies under MDR or equivalent frameworks [85,104].

To illustrate, consider a regional healthcare authority deploying an IoMT platform for chronic heart failure monitoring. Patients are equipped with wearable ECG patches and smart weight scales, which connect via Bluetooth to a secure home gateway. Edge analytics detect early signs of fluid retention, triggering automated alerts through a standardized HL7 FHIR interface to the regional EHR. A cloud-based decision-support engine aggregates data across facilities, applying population-level risk stratification models. The architecture combines local processing for urgent, patient-specific responses with centralized intelligence for longitudinal care optimization. This hybrid approach minimizes latency in urgent cases, reduces network load, and respects data residency requirements by keeping identifiable patient data within the jurisdiction.

Finally, infrastructural decisions must be context-sensitive: urban hospitals may prioritize high-throughput interoperability with existing enterprise IT systems, whereas rural or resource-constrained settings benefit more from low-power, autonomous edge devices with intermittent connectivity support. Future research should quantify these trade-offs through comparative evaluations, assessing clinical safety, total cost of ownership, cybersecurity resilience, and patient acceptability across deployment contexts.

### 5.3. Validation and Regulatory Readiness

The translation of IoMT technologies from experimental prototypes to fully regulated clinical solutions necessitates rigorous validation processes and robust regulatory alignment. Without these, technologies remain confined to pilot studies and fail to reach large-scale, sustainable adoption within public healthcare systems. Validation is not merely a technical process of confirming device performance under ideal conditions; it involves evaluating clinical utility, patient outcomes, usability, and real-world effectiveness across diverse populations and settings [105].

In line with the European Medical Device Regulation or EU MDR 2017/745 [104], local health authorities require that all IoMT interventions used in patient care undergo clinical evaluation, including evidence from randomized or quasi-experimental trials, usability studies, and post-market surveillance strategies. Moreover, local health authorities promote capacity-building for regulatory literacy. Health professionals often lack training on digital conformity assessment or post-market monitoring obligations. In response, these authorities offer Continuing Medical Education (CME) courses on device certification processes, MDR compliance, and cybersecurity frameworks for health technologies.

### 5.4. Training and Digital Literacy

The long-term integration of IoMT technologies into healthcare ecosystems fundamentally relies on the digital readiness of both professionals and patients. Training programs, onboarding processes, and structured digital literacy interventions are indispensable in mitigating cognitive overload, fostering acceptance, and ensuring the safe, ethical, and effective use of these technologies. Empirical studies [81] demonstrate that while up to 60% of healthcare workers report high self-assessed digital competence, nearly 20% express apprehension regarding clinical implementation, revealing a critical gap between technical familiarity and operational confidence. As for the population, tailored patient education strategies—ranging from multimedia guides to peer-assisted learning—can enhance engagement and reduce technostress, particularly among older or marginalized populations. These interventions not only support the responsible use of IoMT devices but also contribute to improved health outcomes, safety, and equity. Arias López and colleagues define digital literacy as “a new determinant of health” [106]. In a systematic review in 2022 [107], Tinmaz et al. highlighted the wide disparities in digital literacy between regions. Digital inequalities include skills, access, usage, and self-perception. These inequalities need to be addressed, as they are considered to have ‘the potential to shape life opportunities in multiple ways’ [108]. For example, educational achievement, labour market competitiveness, health, civic, and political participation. We should not forget that digital inequalities have increased due to the COVID-19 pandemic and have affected the very health status of the most vulnerable population groups or their employability at a time when digital skills are in demand [109,110]. As demonstrated by models such as EMR-integrated literacy assessments and participatory design frameworks [111], digital literacy must be viewed not as a peripheral concern, but as a strategic axis of healthcare transformation. Embedding these components into institutional governance ensures a sustainable and human-centered digital transition.

## 6. Axis 4: Equity, Sustainability, and Governance Analysis

### 6.1. Equity and Accessibility

The deployment of IoMT technologies in healthcare raises significant equity and accessibility considerations, particularly when embedded within universal public health systems. Equity in digital health extends beyond mere access to technology; it encompasses the capacity to meaningfully engage with and benefit from it [112]. Local health authorities have developed equity frameworks focusing on three core dimensions: digital literacy, infrastructural access, and cultural responsiveness. These dimensions are operationalized through targeted interventions designed to close the digital divide and ensure that underserved populations, particularly older adults, migrants, and those with chronic illnesses, are not left behind in the transition to digitally augmented care.

Multilingual training sessions, simplified user interfaces, and in-home technical support provided by community health workers are some of the solutions that are used daily. Geographic equity is also a priority. Remote areas often suffer from inadequate broadband infrastructure, a prerequisite for many IoMT systems. In order to reach marginalized populations without internet access, USL TC has deployed hybrid models combining IoMT with traditional community health strategies. In such models, digital tools are complemented by analog outreach—home visits, paper-based education, and telephone follow-ups—ensuring that benefits are not confined to the digitally literate alone [113]. In terms of structural policy, USL TC aligns with the WHO Global Strategy on Digital Health 2020–2025 [114], which emphasizes “leaving no one behind” as a guiding principle.

### 6.2. Long-Term Engagement and Adoption

Sustained user engagement is one of the most decisive factors in the long-term success IoMT interventions [75]. While initial adoption may be driven by novelty or external incentives, enduring participation requires continuous perceived value, ease of use, trust in the system, and integration into daily routines. A major impediment to long-term engagement is the drop-off in adherence to monitoring protocols over time. Research consistently shows that without personalized support or system adaptability, patient participation in remote monitoring declines significantly after the first three months [115]. Behavioural economics has further informed the engagement design adopted by several local health authorities. Concepts such as “present bias” (the cognitive tendency to overvalue immediate rewards at the expense of long-term benefits) and “loss aversion” (the psychological phenomenon whereby individuals tend to prefer avoiding losses rather than acquiring equivalent gains) have been operationalized through goal-setting dashboards and reward systems that reinforce consistent usage [116].

### 6.3. Economic Considerations and Sustainability

Economic viability is a critical determinant of the long-term success of IoMT initiatives, especially within tax-funded public health systems [75]. While initial investments often focus on proof-of-concept or pilot implementation, sustainable scale-up requires evidence of cost-effectiveness, return on investment (ROI), and optimized resource allocation. Traditional health economic models, such as cost–utility and cost–benefit analyses, have been adapted to accommodate dynamic, real-time data flows typical of IoMT systems. For example, a multicentre wearable sensor system predicted impending heart failure hospitalizations with 76–88% sensitivity and 85% specificity (LINK-HF study) [117]. Operational efficiencies are also crucial. IoMT systems can reduce human resource expenditures by automating routine measurements, triaging cases based on real-time data, and minimizing unnecessary in-person visits [118]. Beyond direct cost savings, indirect economic benefits must also be considered. These include patient productivity, caregiver burden reduction, and long-term health system resilience [119].

### 6.4. Policy and Governance Recommendations

The integration of IoMT into healthcare systems presents both a policy opportunity and a governance imperative. As digital health solutions become increasingly embedded in care delivery, the regulatory, ethical, and administrative frameworks surrounding them must evolve accordingly. The experience of different local health authorities illustrates how sub-national healthcare systems can develop forward-looking governance models that align with broader policy goals while remaining responsive to local healthcare needs. The first and most important recommendation is the alignment with emerging EU-level instruments, including the Artificial Intelligence Act [120] and the European Health Data Space, or EHDS [121], to provide clear guidance for classification, certification, and post-market monitoring of IoMT systems [122]. In terms of organizational governance, the creation of multidisciplinary digital health steering committees within health institutions is strongly recommended. These bodies should include clinicians, IT professionals, patients, legal experts, and ethicists, facilitating the co-development of protocols, risk assessments, and data governance policies [123]. Another governance recommendation is to operationalize digital literacy as a governance concern. The success of IoMT systems is contingent on the capacity of both providers and patients to interpret and act on digital information.

## 7. Practical Application of the Framework: A Case Study Example

### 7.1. Case Study Overview

To better illustrate the framework proposed in this paper, we refer to our previous study focused on the integration of Extended Reality (XR) and ICT into therapeutic contexts, aimed at enhancing remote artistic and recreational engagement for physically impaired and socially isolated individuals [60]. The study concentrated on the pre-implementation phase, assessing the levels of knowledge and acceptance of XR technologies among healthcare professionals and the general population. The findings enabled the preliminary definition of user requirements and the development of application scenarios designed to support psycho-physical well-being and improve therapeutic effectiveness, ultimately contributing to a better quality of life for patients. We consider this case particularly relevant, as it demonstrates the potential of integrating innovative digital technologies into existing care pathways to promote inclusion, engagement, and emotional support for vulnerable populations.

### 7.2. Methodological Approach and Tools

To translate into practice the four foundational axes of the framework, we adopted a pragmatic evidence-elicitation workflow designed to populate the movable SWOT analysis with context-specific inputs prior to the introduction of IoMT solutions. Multiple instruments can be used for this purpose, including literature mapping, context analysis, stakeholder elicitation, and routine data extraction. In our case, as exemplified in [60], we utilized a combination of structured questionnaires, focus groups, and expert consultations to (a) establish a baseline regarding knowledge, acceptance, and perceived benefits and risks; (b) identify organizational workflows and constraints; and (c) elicit ethical, regulatory, and governance considerations. These materials fed the four axes of the framework and the resulting context-aware matrix served to guide pre-implementation assessments and the design of scalable, user-centered deployments. Furthermore, it facilitated the generation of RWD to support subsequent value demonstration and justification of expenditures.

### 7.3. Framework Application and Results

Building on the definition of user requirements and application scenarios, we first identified XR technologies as the central component of our system for delivering immersive experiences to individuals with physical impairments and social isolation (*Axes 1*). XR technologies can be complemented by wearable and non-contact sensors for real-time monitoring of emotional and physiological responses, and by AI algorithms capable of detecting patterns and adapting content to individual needs. However, several key obstacles to XR adoption emerged (*Axes 2*). Technologically, ensuring interoperability across devices and managing real-time, sensor-based monitoring while safeguarding data privacy remains complex. Organizationally, XR applications need to be integrated into clinical workflows and therapeutic routines, yet limited experience among healthcare professionals and the need for targeted training can hinder adoption. Ethically, continuous data collection raises concerns around informed consent and accountability. From a human-centered perspective, usability is critical, especially for users with low digital literacy or physical limitations. To address these barriers, we suggested a strategy-oriented approach (*Axes 3*), emphasizing participatory design and interdisciplinary collaboration among clinicians, engineers, and data scientists to align technical components with therapeutic goals. The system needs to be developed with inclusive, intuitive interfaces tailored to diverse user profiles, supported by targeted training programs to foster digital literacy. Infrastructural considerations included the integration of lightweight, energy-efficient sensors and interoperable device architectures, ensuring both adaptability and economic sustainability. Finally, in the context of our case study, ensuring economic sustainability required comprehensive logistical planning and active collaboration among stakeholders to guarantee that the system could meet therapeutic needs without becoming prohibitively expensive (*Axes 4*).

## 8. Conclusions

This paper has explored the critical transition from technological innovation to the systemic integration of IoMT within real-world healthcare ecosystems. Anchored in four thematic axes, the present study offers a multidimensional framework to address this complex transformation. First, a structured analysis of the current technological landscape has identified the foundational components and demonstrated capabilities of IoMT systems, emphasizing their potential to enhance monitoring, personalization, and care coordination. Second, by systematically classifying integration barriers—spanning technological, organizational, ethical, and human-centered domains—the study revealed the multidimensional challenges that must be addressed to achieve sustainable adoption. Third, strategic enablers—such as participatory design, digital literacy, regulatory alignment, and interoperable infrastructures—were outlined to foster ethically grounded, context-aware, and scalable deployment models. Finally, particular attention was devoted to ensuring that integration processes promote equity, long-term user engagement, economic sustainability, and adaptive governance. Together, these contributions present an operational roadmap to guide future research and policy efforts. Bridging the gap between IoMT innovation and healthcare practice requires not only technical excellence but also interdisciplinary collaboration, inclusive design, and robust governance mechanisms. Only through such integrative approaches can IoMT realize its transformative promise across diverse healthcare contexts.

## Figures and Tables

**Table 1:** Barriers to IoMT integration in healthcare systems.

Category	Identified Barriers	Impacts
Technological	Lack of interoperability, siloed systems, limited AI explainability, weak edge AI validation, poor system adaptability	Limited scalability and integration, workflow disruptions, reduced decision accuracy
Organizational	Rigid workflows, lack of training, cognitive overload	Low adoption, clinician burnout
Ethical/Regulatory	Privacy, explainability, data protection issues	Legal risks, mistrust, inequity
Human-Centered	Device discomfort, low digital literacy	Low adherence, user dropout

**Table 2:** Enabling strategies for the real-world integration of IoMT systems.

Area	Key Strategy or Tool	Description
Contextual Adaptation and Participatory Design	Alignment with clinical workflows, co-design with end-users	Ensures contextual fit, cultural sensitivity, and user acceptance of IoMT system
Technology and Infrastructure	Edge–cloud architectures, HL7 FHIR middleware	Enables low-latency, secure, and interoperable data management across platforms
Governance and Oversight ^(*)^	Multidisciplinary health boards, digital steering committees	Facilitates coordination, accountability, and alignment with health policies
Training and Digital Readiness	Continuing education, onboarding programs	Builds capacity among professionals and patients to engage with digital tools
Regulation and Validation	Clinical evaluation, MDR compliance, explainability frameworks	Ensures reliability, legal compliance, and trustworthy use of IoMT
Equity and Accessibility ^(**)^	Hybrid models, multilingual support, outreach strategies	Addresses the digital divide and promotes inclusion of underserved populations

^(*)^ Governance-related considerations are addressed in greater detail in Section 6.4. ^(**)^ Equity-related considerations are addressed in greater detail in Section 6.1.

## Data Availability

Data is contained within the article.
